# Common P-glycoprotein (*ABCB1*) polymorphisms do not seem to be associated with the risk of rivaroxaban-related bleeding events: Preliminary data

**DOI:** 10.11613/BM.2024.020703

**Published:** 2024-04-15

**Authors:** Ana Marija Slišković, Jozefina Palić, Tamara Božina, Lana Ganoci, Majda Vrkić Kirhmajer, Vladimir Trkulja, Joško Bulum, Livija Šimičević

**Affiliations:** 1 Department of Cardiovascular Diseases, University Hospital Centre Zagreb, Zagreb, Croatia; 2 Department of Medical Chemistry, Biochemistry and Clinical Chemistry, University of Zagreb School of Medicine, Zagreb, Croatia; 3 Division of Pharmacogenomics and Therapy Individualization, Department of Laboratory Diagnostics, University Hospital Centre Zagreb, Zagreb, Croatia; 4 Department of Internal Medicine, University of Zagreb School of Medicine, Zagreb, Croatia; 5 Department of Pharmacology, University of Zagreb School of Medicine, Zagreb, Croatia

**Keywords:** adverse drug reactions, cardiovascular disease, hemorrhage, pharmacogenetics, risk factors, rivaroxaban

## Abstract

**Introduction:**

Considering conflicting previous reports, we aimed to evaluate whether the common *ABCB1* polymorphisms (rs1128503, rs2032582, rs1045642, rs4148738) affected the risk of bleeding in rivaroxaban-treated patients.

**Materials and methods:**

We report preliminary data from a larger nested case-control study. Consecutive adults started on rivaroxaban for any indication requiring > 6 months of treatment were followed-up to one year. Patients who experienced major or non-major clinically relevant bleeding during the initial 6 months were considered cases, whereas subjects free of bleeding over > 6 months were controls. The polymorphisms of interest (rs1128503, rs2032582, rs1045642, rs4148738) were in a strong linkage disequilibrium, hence patients were classified regarding the “load” of variant alleles: 0-2, 3-5 or 6-8. The three subsets were balanced regarding a range of demographic, comorbidity, comedication and genetic characteristics. A logistic model was fitted to probability of bleeding.

**Results:**

There were 60 cases and 220 controls. Raw proportions of cases were similar across the subsets with increasing number of *ABCB1* variant alleles (0-2, N = 85; 3-6, N = 133; 6-8, N = 62): 22.4%, 21.8%, and 19.4%, respectively. Fully adjusted probabilities of bleeding were also similar across the subsets: 22.9%, 27.5% and 17.7%, respectively. No trend was observed (linear, t = -0.63, df = 273, P = 0.529; quadratic, t = -1.10, df = 273, P = 0.272). Of the 15 identified haplotypes, the completely variant (c.1236T_c.2677T(A)_c.3435T_c.2482-2236A) (40.7%) and completely wild-type (C_G_C_G) (39.5%) haplotypes prevailed, and had a closely similar prevalence of cases: 21.1% *vs*. 23.1%, respectively.

**Conclusions:**

The evaluated common *ABCB1* polymorphisms do not seem to affect the risk of early bleeding in patients started on rivaroxaban.

## Graphical abstract

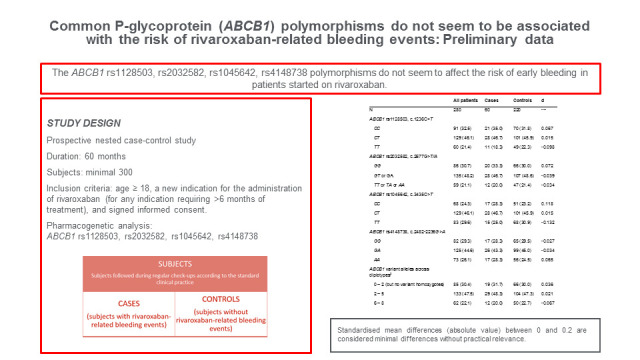

## Highlights

• An evaluation of the association of the common *ABCB1* polymorphisms and risk of bleeding in rivaroxaban-treated patients was performed

• The four common *ABCB1* polymorphisms did not affect the risk of bleeding in rivaroxaban-treated patients of European descent

• Consistent results were observed based on raw data and fully adjusted data, in the analysis based on the “load” of variant alleles across the four polymorphisms, haplotype-based analysis and analysis based on the combination of genotypes at the investigated polymorphisms

## Introduction

Rivaroxaban, a factor Xa inhibitor, is the most prescribed of the non‐vitamin K antagonist oral anticoagulants (NOACs), a class of drugs that is progressively replacing classical oral anticoagulants (vitamin K antagonists, VKA) worldwide, in a wide range of indications (*1*-*3*). As is the case with other NOACs (Xa inhibitors apixaban and edoxaban, and a direct thrombin inhibitor dabigatran), rivaroxaban is favored over VKAs for simplicity of use that does not require constant monitoring of the coagulation cascade and, generally, for a lower risk of major bleedings (*4*). However, exposure to and anticoagulant effect of rivaroxaban is affected by age, sex, body weight, hepatic and renal function, concomitant diseases and treatments (*5*, *6*).

Interactions between rivaroxaban and other drugs are typically pharmacokinetic and are based on the fact that rivaroxaban is a substrate of cytochrome P-450 enzymes CYP3A4/5 and CYP2J2 (and several CYP-independent mechanisms), as well as of two major efflux transporter proteins – ABCB1 (multidrug resistance protein 1, MDR1 or P-glycoprotein, P-gp) and ABCG2 (breast cancer resistance protein, BCRP) (*7*, *8*). There is some evidence that polymorphisms in genes encoding the respective metabolizing enzymes and/or transporter proteins (*CYP3A4*, *CYP3A5*, *CYP2J2*, *ABCB1*, *ABCG2*) might affect exposure to and efficacy/safety of rivaroxaban, but at present, the results are equivocal and insufficient for implementation in clinical practice (*9*-*11*).

ABCB1 protein is a crucial efflux membrane transporter with a protective function, and with a wide range of endogenous and exogenous substrates, including many drugs. Located at the apical membrane, it excretes orally administered drugs back into the small intestine and colon, in the kidney it excretes substances into the tubular lumen, and in the liver it excretes them into the bile, thus reducing their circulating levels (*12*-*14*). Although there is evidence for interindividual differences in the ABCB1 expression and transport function, the genetic contribution is still not fully understood (*15*-*17*). It has been implied that *ABCB1* rs1128503, rs2032582 and rs1045642 polymorphisms reduce the ABCB1 transport function *in vitro*, but that the effect might be substrate-dependent (*18*). The fundamental *in vitro* research concerning the association of rivaroxaban with the ABCB1 transport function was published 10 years ago – cellular efflux of rivaroxaban was markedly reduced by two ABCB1 inhibitors, whereas its clearance was greatly reduced in knock-out mice lacking ABCB1 (and ABCG2) transporters (Mdr1a/Mdr1b(-/-)/Bcrp(-/-) mice) (*19*, *20*). The *ABCB1* gene is highly polymorphic. The most common (particularly in European population) and the most extensively investigated *ABCB1* polymorphisms are three coding polymorphisms: rs1128503 (*ABCB1* c.1236C>T); rs2032582 (*ABCB1* c.2677G>T/A) and rs1045642 (*ABCB1* c.3435C>T) (*21*). These polymorphisms are in a strong linkage disequilibrium (LD), and haplotypes with variant alleles (*e.g.*, TTT) – compared to wild type (CGC) – are associated with altered ABCB1 protein folding, binding site conformation and reduced transporter activity *in vitro* (*11*, *22*, *23*). Among other *ABCB1* polymorphism, rs4148738 (*ABCB1* c.2482-2236G>A) is relatively common in European population (*21*). This intronic polymorphism was also found to affect the pharmacokinetics of ABCB1 substrates (*11*). The first indication that the *ABCB1* c.3435C>T (rs104566642) and c.2677G>T (rs2032582) polymorphisms might be associated with the bleeding risk in rivaroxaban-treated patients was based on a case report (*24*). Therefore, it is plausible to assume that these four common *ABCB1* polymorphisms – through the resulting reduced transporter activity – might increase bioavailability of rivaroxaban. This, in turn, could reflect on its anticoagulant activity. Consequently, we aimed to estimate whether they affected the risk of bleeding in rivaroxaban-treated patients.

## Materials and methods

### Subjects and study design

Presented data are part of a larger prospective nested case-control study (“Pharmacogenomics in Prediction of Cardiovascular Drugs Adverse Reaction”) that started December 15, 2020 and will last 60 months and include 1200 subjects. The study (ClinicalTrials.gov, NCT05307718) is conducted in accordance with the Declaration of Helsinki and was approved by the Ethics Committees of the University of Zagreb, School of Medicine (reg. number 380-59-10106-20-111/125; class 641-01/20-02/01) and the University Hospital Centre Zagreb (class 8.1-20/142-2; number 02/21 AG), Zagreb, Croatia. The primary cohort includes adults (> 18 year of age) with a new-onset indication for treatment with NOACs, antiplatelets and/or statins. All subjects provided written informed consent.

The present analysis included consecutive consenting adults (≥ 18 years of age) who started rivaroxaban for any indication requiring > 6 months of therapy, and were followed-up until occurrence of bleeding, or if no bleeding occurred, for > 6 up to 12 months within the time period between December 15, 2020 and March 1, 2023.

Comedication present at baseline or in place for at least one month before the bleeding event in cases, or in place for at least (any) 3 months in controls, was classified as (enzyme or transporter) substrates, inducers or inhibitors using the Lexicomp Clinical Decision Support System (*25*).

Patients who developed major or clinically relevant non-major bleeding, as defined by the International Society on Thrombosis and Haemostasis (ISTH), within the first 6 months of treatment were considered cases, whereas controls were patients who experienced no bleeding over > 6 months of treatment (*26*, *27*).

Major bleeding is defined as fatal bleeding, and/or symptomatic bleeding in a crucial area or organ, and/or bleeding resulting with a decrease in hemoglobin concentrations of ≥ 20 g/L, or indicating a transfusion of ≥ 2 units of whole blood or red cells (*26*). Non-major bleeding is defined as multiple-source bleeding, unexpected hematoma (> 25 cm^2^), epistaxis (> 5 minutes), gingival bleeding (> 5 minutes), macroscopic hematuria, rectal bleeding, coughing or vomiting blood, vaginal bleeding, blood in semen, intra-articular bleeding with trauma, or surgical-site bleeding (*27*).

All present subjects were recruited at a single tertiary centre. They were treated and followed-up in line with the standard of care by physicians specialized in management of the respective conditions (*e.g.*, non-valvular atrial fibrillation, deep vein thrombosis, pulmonary embolism, secondary prophylaxis after acute coronary syndromes, primary prophylaxis in patients with coronary artery disease or peripheral artery disease). Patients were instructed to contact their prescribing physician regardless of their regular scheduled visits in case of any bleeding, which was then assessed in line with the ISTH criteria (*26*, *27*). Attending physicians were not aware of the patients’ pharmacogenetic status at the time of assessment of severity of bleeding or confirmation of the “control” status. The “control” status for patients not experiencing bleeding over > 6 months of treatment was verified by telephone contacts at the cut-off date defined for the purpose of the present analysis.

### Blood sampling

At the inception of the cohort, blood samples were taken for the genetic analysis (3 mL of whole blood) in an K_3_EDTA tube (Vacuette, Greiner Bio-One International AG, Kremsmünster, Austria). For routine biochemical, hematological and coagulation analyses following blood samples were taken: 8 mL of serum in a test tube without biochemical additives (Vacuette, Greiner Bio-One International AG, Kremsmünster, Austria) and/or 3 mL of whole blood in a K_3_EDTA tube (Vacuette, Greiner Bio-One International AG, Kremsmünster, Austria) and/or 2.7 mL of plasma in a sodium citrate tube 0.105 M (3.2%) (Becton Dickinson, Plymouth, United Kingdom), respectively. Routine biochemical, hematological and coagulation analyses were performed according to the attending physicians’ orders.

### Isolation of DNA and pharmacogenetic analyses

Genomic DNA was extracted from whole blood using the QIAamp DNA Mini Kit (Qiagen, Hilden, Germany), according to the manufacturer’s instructions. Pharmacogenetic analyses were performed by using specific TaqMan DME and SNP Assays on 7500 Real-Time PCR System (Thermo Fisher Scientific, Waltham, USA) for genotyping of *CYP2J2*, *CYP3A4*, *CYP3A5*, *ABCB1*, and *ABCG2* gene variants (*28*). Only for the *ABCB1* triallelic locus c.2677G>T/A (rs2032582) genotyping was performed by the real-time PCR on the LightCycler v 2.0 device (Roche Diagnostics, Germany), as described by von Arjomand-Nahad *et al.* (*29*).

We investigated four *ABCB1* polymorphisms of primary interest: rs1128503 (assay ID C___7586662_10), rs2032582, rs1045642 (assay ID 4362691 C___7586657_20) and rs4148738 (assay ID C___1253813_10). We determined additional gene polymorphisms with possible effect on rivaroxaban pharmacokinetics, and considered them as covariates (confounders): the common loss-of-function *ABCG2* polymorphism c.421C>A, rs2231142 (assay ID 4362691 C__15854163_70), *CYP3A4* *1B, rs2740574 (assay ID 4362691 C___1837671_50), and *22; rs35599367 (assay ID 4351379 C__59013445_10) and *CYP3A5* *3, rs776746 (assay ID C__26201809_30) polymorphisms needed to determine *CYP3A4*/5 genotype-predicted phenotype, and *CYP2J2* A>T (rs11572325, assay ID C__30760106_10) and *7 (rs890293, assay ID 4362691 C___9581699_80) polymorphisms to determine CYP2J2 phenotype. CYP3A4/5 genotype-predicted phenotype is defined as follows: extensive metabolizer – high activity (CYP3A4*1/*1 and CYP3A5*1 carriers); intermediate metabolizer – intermediate activity (CYP3A4*1/*1 or CYP3A4*22 carriers and CYP3A5*3/*3 or CYP3A5*1 carriers) and poor metabolizer – low activity (CYP3A4*22 carriers and CYP3A5*3/*3) (*30*). CYP2J2 genotype-predicted phenotype is defined as high activity (*CYP2J2* *1/*1) and intermediate or low activity (*CYP2J2* *7 carriers) (*31*, *32*).

### Statistical analysis

We expected the four *ABCB1* polymorphisms to be in a strong pairwise LD, and we planned to classify the subjects based on the “load” of variant alleles across them as: i) 0-2 variant alleles, but no locus is variant homozygous (*i.e.*, wild-type or a maximum of 2 heterozygous loci); ii) 2-5 variant alleles, *i.e*, at least one variant homozygous locus to a maximum of 5 variant alleles (2 variant + 1 heterozygous or 1 variant + 3 heterozygous loci); iii) 6-8 variant alleles (*17*, *20*, *25*-*28*). We further expected that distribution of patients across these categories would be 30%, 50% and 20%, respectively, and that 15-20% would experience bleeding over the first 6 months of treatment (*18*, *33*). We approximated that with such prevalence of genotypes and events, a sample of 250-300 patients would provide 80-85% probability to detect a strong, clear increasing trend (the theoretical background suggests that variant alleles result in reduced ABCB1 function) in proportion of cases across the levels of *ABCB1* variant alleles (*e.g.*, 10%-20%-30%) (*34*).

We used energy balancing with average treatment effect as the estimand (package WeightIT in R Statistical Software, v4.1.2, R Core Team 2021) to achieve a balance between cases and controls regarding a number of demographic, comorbidity, (co)medication and pharmacogenetic variables (except for the *ABCB1* polymorphisms) that could have confounded the relationship between the polymorphisms of interest and the case/control status (*35*, *36*). Energy balancing is a weighting method that achieves (where possible) a distributional balance of covariates between groups (*37*, *38*). Standardized differences (d) < 0.1 indicate an adequate balance, *i.e*., irrelevant differences. Sporadic covariates that could not be adequately balanced (d ≥ 0.1) were included in a multivariable weighted logistic regression model with robust variance estimation to generate estimated (adjusted) probabilities of bleeding. We report: a) raw and weighted (after energy balancing) proportions of patients with different “load” of *ABCB1* variant alleles in cases and controls; b) raw, weighted (after energy balancing) and fully adjusted (from the logistic model) proportion of cases across the patient subsets with different “load” of *ABCB1* variant alleles, and the associated test for trend in proportions. To supplement this analysis, we report also prevalence of all identified halplotypes and genotypes, and raw proportions of “cases” (incidence of bleeding) across them. We used SAS for Windows 9.4 (SAS Inc., Cary, USA). We used webtool CubeX (http://apps.biocompute.org.uk/cubex) to determine Hardy-Weinberg equilibrium and LD (*39*).

## Results

There were a total of 60 cases and 220 control patients (Table 1). Cases were older (mean 73 *vs*. 63 years, d = 0.799) and had somewhat lower estimated glomerular filtration rate (eGFR) (mean 65 *vs*. 74 mL/min/1.73 m^2^, d = - 0.372). Three of the case patients experienced a major bleeding (2 intracranial, 1 hemopericardium), while the rest were non-major bleedings, predominantly gastrointestinal. Rivaroxaban doses and co-treatment with antiplatelets were fairly similar in cases and controls (all d < 0.300). Cases and controls somewhat differed in prevalence of major comorbidities, with a clear difference in prevalence of malignant diseases (35% *vs*. 15.9%, d = 0.449) and gastrointestinal diseases (typically peptic acid disease and/or diverticulosis; 46.7% *vs*. 6.8%, d = 1.008) (Table 1).

**Table 1:** Demographics and comorbidities for patients, cases and controls

	**All patients** **(N = 280)**	**Cases** **(N = 60)**	**Controls** **(N = 220)**	**d**
Age (years)	65 (26-98)	73 (30-88)	63 (26-98)	0.799
Men (N,%)	172 (61)	33 (55)	139 (63)	- 0.167
eGFR (mL/min/1.73 m^2^)	72 ± 22	65 ± 25	74 ± 25	- 0.372
**Bleeding (N,%)**				
Major – hemopericardium	1 (0.35)	1 (1.67)	/	/
Major – intracranial	2 (0.71)	2 (3.33)	/	/
Non-major, relevant	57 (20.3)	57 (95.0)	/	/
Gastro-intestinal	27 (9.6)	27 (45.0)	/	/
Nosebleed	10 (3.6)	10 (16.7)	/	/
Hematuria	8 (2.9)	8 (13.3)	/	/
Hematoma	3 (1.1)	3 (5.0)	/	/
All other locations	9 (3.3)	9 (15.0)	/	/
**Rivaroxaban daily dose (mg) (N,%)**				
1 x 20 mg	202 (72.1)	40 (66.7)	162 (73.6)	- 0.153
1 x 15 mg	15 (12.9)	11 (18.3)	25 (11.4)	0.197
1 x 10 mg	10 (3.6)	5 (8.3)	5 (2.3)	0.273
2 x 2.5 mg	32 (11.4)	4 (6.7)	28 (12.7)	- 0.206
Co-treated with antiplatelets	61 (21.8)	14 (23.3)	47 (21.4)	0.047
Low dose aspirin	45 (16.1)	10 (16.7)	35 (15.9)	0.021
Clopidogrel	26 (9.3)	6 (10.0)	20 (9.1)	0.031
Non-valvular atrial fibrillation	201 (71.8)	46 (76.7)	155 (70.5)	0.141
Venous thromboembolism	39 (13.9)	12 (20.0)	27 (12.3)	0.211
Hypertension	236 (84.3)	54 (90.0)	182 (82.7)	0.213
Diabetes mellitus	66 (23.6)	13 (21.7)	53 (24.1)	- 0.058
Thyroid gland disease	56 (20.0)	10 (16.7)	46 (20.9)	- 0.109
Dyslipidemia	184 (65.7)	37 (61.7)	147 (66.8)	- 0.108
Cardiovascular incidents	100 (35.7)	21 (35.0)	79 (35.9)	- 0.019
Ischemic stroke / TIA	42 (15.0)	6 (10.0)	36 (16.4)	- 0.189
Coronary syndrome/AMI	67 (23.9)	17 (28.3)	50 (22.7)	0.129
Peripheral artery disease	47 (16.8)	12 (20.0)	35 (15.9)	0.107
Adult congenital heart disease	5 (1.8)	0	5 (2.3)	/
Valvular disease/surgery	10 (3.6)	3 (5.0)	7 (5.9)	0.092
Antiphospholipid syndrome	16 (5.7)	3 (5.0)	13 (5.9)	- 0.040
History of malignancy	56 (20.0)	21 (35.0)	35 (15.9)	0.449
Solid organ cancer	45 (16.1)	18 (30.0)	27 (12.3)	0.445
Hematological malignancy	14 (5.0)	4 (6.7)	10 (4.6)	0.092
Autoimmune disease	10 (3.6)	5 (8.3)	5 (2.3)	0.273
Rheumatoid arthritis	8 (2.9)	3 (5.0)	5 (2.3)	0.146
Other autoimmune diseases	2 (0.7)	2 (3.3)	0	/
COPD or asthma	13 (4.6)	2 (3.3)	11 (5.0)	- 0.080
Gastrointestinal diseases	43 (15.4)	28 (46.7)	15 (6.8)	1.008
Age is presented as median (range). eGFR is presented as mean and standard deviation. Cases - patients experiencing bleeding within the first 6 months of treatment. Controls - patients treated for > 6 months, not experiencing bleeding. d - standardized mean differences (cases *vs.* controls): 0-2 – minimal, without practical relevance; ≥ 0.2 to 0.5 or 0.6 – moderate; > 0.5 or > 0.6 - large differences. Gastrointestinal diseases comprised one patient with inflammatory bowel disease, others - peptic acid disease and/or diverticulosis. AMI – acute myocardial infarction. COPD – chronic obstructive pulmonary disease. eGFR – estimated glomerular filtration rate. TIA – transitory ischemic attack.				

Prevalence of genotypes across the four *ABCB1* polymorphisms was closely similar in cases and controls (all d < 0.2) (Table 2). In line with the expectations, polymorphisms were in a strong pairwise LD (rs1128593 *vs.* rs2032582 D’ = 0.878, r^2^ = 0.750, Chi^2^ = 209.9; rs1128593 *vs.* rs1045642 D’ = 0.890, r^2^ = 0.574, Chi^2^ = 164.2; rs1128593 *vs.* rs4148738 D’ = 0.892, r^2^ = 0.680, Chi^2^ = 190.4; rs2032582 *vs.* rs1045642 D’ = 0.876, r^2^ = 0.561, Chi^2^ = 157.1; rs2032582 *vs.* rs4148738 D’ = 0.725, r^2^ = 0.331, Chi^2^ = 144.0; rs1045642 *vs.* rs4148738 D’ = 0.732, r^2^ = 0.469, Chi^2^ = 143.1), hence subjects were classified as planned, based on the “load” of variant alleles across the four loci as those with 0-2 variant alleles, but no variant homozygous locus (reference group), those with 2-5 variant alleles and those with 6-8 variant alleles: distribution of cases and controls across these three groups was closely similar (all d < 0.1). Prevalence of patients using different numbers of ABCB1 substrates or inductors was closely similar in cases and controls (all d < 0.1) (Table 2). Only the proportions of patients using 2 or ≥ 3 ABCB1 inhibitors were slightly different in cases than in controls (41.7% *vs*. 31.4%, d = 0.215, and 33.3% *vs*. 39.5%, d = -0.129, respectively) (Table 2).

**Table 2:** Genotypes at *ABCB1* polymorphisms of interest and use of ABCB1 substrates, inhibitors and inducers

	**All patients** **(N = 280)**	**Cases** **(N = 60)**	**Controls** **(N = 220)**	**d**
***ABCB1* rs1128503, c.1236C*>T* (N,%)**				
CC	91 (32.5)	21 (35.0)	70 (31.8)	0.067
CT	129 (46.1)	28 (46.7)	101 (45.9)	0.015
TT	60 (21.4)	11 (18.3)	49 (22.3)	- 0.098
***ABCB1* rs2032582, c.2677G*>T/A* (N,%)**				
GG	86 (30.7)	20 (33.3)	66 (30.0)	0.072
*GT* or GA	135 (48.2)	28 (46.7)	107 (48.6)	- 0.039
*TT* or *TA* or *AA*	59 (21.1)	12 (20.0)	47 (21.4)	- 0.034
***ABCB1* rs1045642, c.3435C*>T* (N,%)**				
CC	68 (24.3)	17 (28.3)	51 (23.2)	0.118
CT	129 (46.1)	28 (46.7)	101 (45.9)	0.015
TT	83 (29.6)	15 (25.0)	68 (30.9)	- 0.132
***ABCB1* rs4148738, c.2482-2236*G>A* (N,%)**				
GG	82 (29.3)	17 (28.3)	65 (29.5)	- 0.027
GA	125 (44.6)	26 (43.3)	99 (45.0)	- 0.034
AA	73 (26.1)	17 (28.3)	56 (24.5)	0.065
***ABCB1* variant alleles across diplotypes (N,%)**				
0–2 (but no variant homozygotes)	85 (30.4)	19 (31.7)	66 (30.0)	0.036
2–5	133 (47.5)	29 (48.3)	104 (47.3)	0.021
6–8	62 (22.1)	12 (20.0)	50 (22.7)	- 0.067
**Number of comedication ABCB1 substrates (N,%)**	2 (1-3)	2 (1-3)	2 (1-3)	/
0–1	87 (31.1)	17 (28.3)	70 (31.8)	- 0.076
2	103 (36.8)	24 (40.0)	79 (35.9)	0.084
≥ 3	90 (32.1)	19 (31.7)	71 (32.3)	- 0.013
**Number of comedication ABCB1 inhibitors (N,%)**	2 (1-3)	2 (1-3)	2 (1-3)	/
0–1	79 (28.2)	15 (25.0)	64 (29.1)	- 0.092
2	94 (33.6)	25 (41.7)	69 (31.4)	0.215
≥ 3	107 (38.2)	20 (33.3)	87 (39.5)	- 0.129
**Comedication ABCB1 inductor (maximum 1) (N,%)**	12 (4.3)	2 (3.3)	10 (4.5)	- 0.062
Cases - patients experiencing bleeding within the first 6 months of treatment. Controls - patients treated for > 6 months, not experiencing bleeding. d - standardized mean differences (cases *vs.* controls): 0-2 – minimal, without practical relevance; ≥ 0.2 to 0.5 or 0.6 – moderate; > 0.5 or > 0.6 - large differences. All four *ABCB1* SNPs were in a strong pairwise LD. There was no departure from HWE for any polymorphism: rs1128593 Chi^2^ = 1.26, P = 0.262; rs2032582 Chi^2^ = 0.199, P = 0.655; rs1045642 Chi2 = 1.61, P = 0.204; rs4148738 Chi^2^=3.16, P = 0.076.				

Cases and controls were closely similar regarding the prevalence of *ABCG2* c.421C>A variant carriers and ABCG2 substrate or inhibitor users (all d < 0.1) (Table 3); *CYP3A4*/*5* polymorphisms and predicted phenotype, as well as the prevalence of CYP3A4/5 substrate users (all d < 0.2), with somewhat more CPY3A4/5 inhibitor users among controls (30.0%) than among (d = 0.232) cases (20.0%). They were also similar regarding prevalence of *CYP2J2* genotypes and predicted phenotype, whereas less cases than controls were using CYP2J2 inhibitors (13.3% *vs*. 25.0%) (d = -0.300) (Table 3).

**Table 3:** Genotypes at the *ABCG2*, *CYP3A4*, *CYP3A5* and *CYP2J2* polymorphisms, genotype-predicted phenotypes and use of substrates, inhibitors and inducers

	**All patients** **(N = 280)**	**Cases** **(N = 60)**	**Controls** **(N = 220)**	**d**
***ABCG2* rs2231142, *c.421C>A* (N,%)**				
CC	231 (82.5)	49 (81.7)	182 (82.7)	- 0.027
CA	47 (16.8)	10 (16.7)	37 (16.8)	- 0.004
AA	2 (0.7)	1 (1.6)	1 (0.5)	0.118
**Comedication ABCG2 substrates (N,%)**	1 (1-2)	1 (1-2)	1 (1-2)	/
0–1	168 (60.0)	37 (61.7)	131 (59.5)	0.043
2–3	112 (40.0)	23 (38.3)	89 (40.5)	- 0.043
**Comedication ABCG2 inhibitor (maximum 1) (N,%)**	188 (67.1)	42 (70.0)	146(66.4)	0.078
CYP3A4*1B (N,%)				
*1/*1	271 (96.8)	60 (100)	211 (95.9)	/
*1/*1B	8 (2.9)	0	8 (3.6)	/
*1B/*1B	1 (0.4)	0	1 (0.5)	/
CYP3A4*22 (N,%)				
*1/*1	264 (94.3)	56 (93.3)	208 (94.5)	/
*1/*22	15 (5.3)	3 (5.0)	12 (5.5)	/
*22/*22	1 (0.4)	1 (1.7)	0	/
CYP3A5*3 (N,%)				
*1/*1	2 (0.7)	0	2 (0.9)	/
*1/*3	33 (11.8)	8 (13.3)	25 (11.4)	/
*3/*3	245 (87.5)	52 (86.7)	193 (87.7)	/
**CYP3A4/5 phenotype (N,%)**				
High activity	34 (12.1)	7 (11.7)	27 (12.3)	- 0.019
Intermediate activity	230 (82.1)	49 (81.7)	181 (82.3)	- 0.016
Low activity	16 (5.7)	4 (6.7)	12 (5.4)	0.051
**Number of comedication CYP3A4/5 substrates (N,%)**	3 (2-4)	3 (2-4)	3 (1-4)	/
0–1	66 (23.6)	11 (18.3)	55 (25.0)	- 0.162
2–3	116 (41.4)	25 (41.7)	91 (41.4)	0.006
≥ 4	98 (35.0)	24 (40.0)	74 (33.6)	0.132
**Comedication CYP3A4/5 inhibitors (1 – 3) (N,%)**	78 (27.9)	12 (20.0)	66 (30.0)	- 0.232
***CYP2J2* rs11572325, *A>T* (N,%)**				
AA	217 (77.5)	47 (78.3)	170 (77.3)	/
AT	60 (21.4)	12 (20.0)	48 (21.8)	/
TT	3 (1.1)	1 (1.7)	2 (0.9)	/
CYP2J2*7 (N,%)				
*1/*1	244 (87.2)	55 (91.7)	189 (85.9)	/
*1/*7	34 (12.1)	4 (6.7)	30 (13.6)	/
*7/*7	2 (0.7)	1 (1.7)	1 (0.5)	/
**CYP2J2 phenotype (N,%)**				
High activity	244 (87.1)	55 (91.7)	189 (85.9)	0.183
Intermediate or low activity	36 (12.9)	5 (8.3)	31 (14.1)	- 0.183
Comedication CYP2J2 inhibitor (maximum 1) (N,%)	63 (22.5)	8 (13.3)	55 (25.0)	- 0.300
Cases - patients experiencing bleeding within the first 6 months of treatment. Controls - patients treated for > 6 months, not experiencing bleeding. d - standardized mean differences (cases *vs.* controls): 0-2 – minimal, without practical relevance; ≥ 0.2 to 0.5 or 0.6 – moderate; > 0.5 or > 0.6 - large differences. There was no departure from HWE for rs2231142 (Chi^2^ = 0.06, P = 0.816); CYP3A4*22 (Chi^2^ = 2.269, P = 0.132) and CYP3A5*3 (Chi^2^ = 0.567, P = 0.452). For CYP3A4*1B Chi^2^ = 9.63, P = 0.002, but this was due only to 1 variant homozygous patient - had this been a heterozygous patient, Chi^2^ = 0.07, P = 0.791. This had no impact on the classification of the predicted phenotypes.				

To estimate the association between the “load” of variant alleles across the four *ABCB1* polymorphisms and the “case status” (bleeding), cases and controls were subjected to energy balancing regarding age, sex, eGFR, rivaroxaban dose, platelet co-treatment, relevant comorbidities, *ABCG2* c.421 genotype, CYP3A4/5 and 2J2 phenotypes, and exposure to enzyme and transporter substrates and inhibitors (Table 4). Excellent balance was achieved (d < 0.1) for almost all covariates except that age (mean 70 *vs*. 67 years, d = 0.298) and proportions of patients with a history of cancer (26.4% *vs*. 21.8%, d = 0.109) and with gastrointestinal diseases (25.0% *vs*. 14.0%, d = 0.279) were still somewhat higher in cases then in controls (Table 4). Also, exposure to CYP2J2 inhibitors was less common among cases (18.3% *vs*. 22.7%, d = -0.172) (Table 4).

**Table 4:** Prevalence of relevant comorbidities, comedication, and *ABCG2* c.421C>A polymorphism, CYP3A4/5 and CYP2J2 phenotypes after covariate balancing

	**Cases** **(N = 60)**	**Controls** **(N = 220)**	**d**
**Variables included in covariate balancing**			
Age (years)	70 ± 12	67 ± 11	0.298
eGFR (mL/min/1.73 m^2^)	71 ± 21	72 ± 20	- 0.043
Men (N, %)	34.0 (58.8)	122.3 (59.8)	- 0.020
**Rivaroxaban dose (mg/day)**			
20 or 15	48.2 (83.4)	171.4 (83.8)	- 0.010
10 or 5	9.6 (16.6)	33.2 (16.2)	0.010
Antiplatelet co-treatment	14.7 (25.4)	46.8 (22.9)	0.062
Atrial fibrillation	43.2 (74.8)	146.0 (71.4)	0.078
Venous thromboembolism	10.5 (18.2)	32.5 (15.9)	0.063
Hypertension	49.5 (85.7)	174.0 (85.1)	0.017
Stroke/TIA or coronary syndrome/AMI	18.6 (32.5)	68.4 (33.4)	- 0.025
History of malignancy	15.3 (26.4)	44.5 (21.8)	0.109
Autoimmune diseases	2.8 (4.8)	7.7 (3.8)	0.045
Gastrointestinal disease	14.5 (25.0)	28.7 (14.0)	0.279
**Number of comedication ABCB1 substrates**			
0–1	16.8 (29.2)	61.8 (30.2)	- 0.023
2	21.4 (37.1)	76.2 (37.3)	- 0.003
≥ 3	19.5 (33.7)	66.6 (32.5)	0.026
**Number of comedication ABCB1 inhibitors**			
0–1	15.1 (26.1)	55.8 (27.3)	- 0.026
2	20.9 (36.2)	69.1 (33.8)	0.051
≥ 3	21.8 (37.7)	79.7 (38.9)	- 0.027
*ABCG2* rs2231142, c.421*C>A* variant allele	10.1 (17.5)	35.9 (17.5)	- 0.000
**Comedication ABCG2 substrates**			
0–1	35.1 (60.7)	122.2 (59.8)	0.018
2–3	22.7 (39.3(	82.4 (40.2)	- 0.018
Comedication ABCG2 inhibitor (maximum 1)	38.9 (67.4)	138.6 (67.8)	- 0.009
**CYP3A4/5 phenotype**			
High activity	7.0 (12.1)	25.6 (12.5)	- 0.012
Intermediate or low activity	50.8 (87.9)	179.0 (87.5)	0.012
**Number of comedication CYP3A4/5 substrates**			
0–1	12.7 (22.0)	48.7 (23.8)	- 0.044
2–3	22.0 (38.0)	82.1 (40.1)	- 0.043
≥ 4	23.1 (40.0)	73.8 (36.1)	0.082
Comedication CYP3A4/5 inhibitors (1 - 3)	15.0 (26.0)	59.1 (28.9)	- 0.069
**CYP2J2 phenotype**			
High activity	51.2 (88.5)	177.6 (86.8)	0.054
Intermediate or low activity	6.6 (11.5)	26.9 (13.2)	- 0.054
Comedication CYP2J2 inhibitor (maximum 1)	10.6 (18.3)	44.4 (22.7)	-0.114
**Not included in covariate balancing**			
*ABCB1* variant alleles across diplotypes			
0–2 (but no variant homozygotes)	17.6 (30.5)	63.1 (30.8)	- 0.007
2–5	31.4 (54.2)	96.6 (47.3)	0.140
6–8	8.8 (15.3)	44.8 (21.9)	- 0.172
Data are presented as weighted means ± SD or weighted proportions (%). Cases - patients experiencing bleeding within the first 6 months of treatment. Controls - patients treated for > 6 months, not experiencing bleeding. d - standardized mean differences (< 0.1 indicated adequate balance, 0-2 – minimal, without practical relevance; ≥ 0.2 to 0.5 or 0.6 – moderate; > 0.5 or > 0.6 - large differences). The effective sample sizes were 57.8 for Cases and 204.6 for the Controls. AMI – acute myocardial infarction. eGFR – estimated glomerular filtration rate. TIA – transitory ischemic attack.			

**Figure 1:**
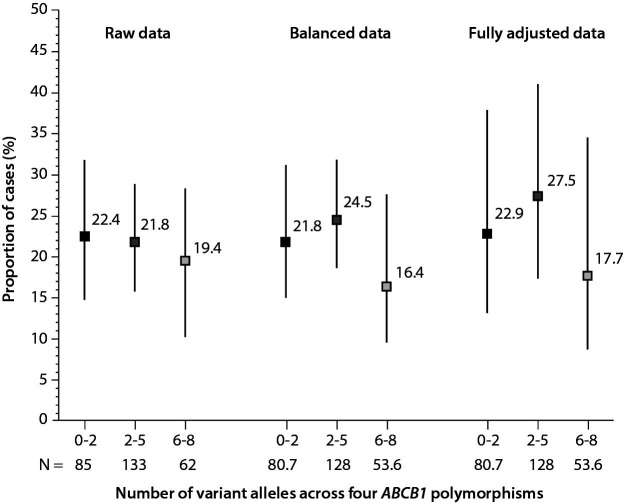
Probability (%) of being a case (bleeding) across the categories of patients with in-creasing number of variant alleles across the four *ABCB1* polymorphisms of interest: 0-2 variant alleles (no variant homozygous loci), 2-5 and 6-8. Probabilities are shown for the raw data (Cochrane-Armitage test for trend z = 0.419, P = 0.338 for an increasing trend, P = 0.662 for a de-creasing trend, P = 0.675 for any trend), for data after covariate balancing (weighted proportions) (Cochrane-Armitage test for trend z = 0.595, P = 0.276 for an increasing trend, P = 0.726 for a de-creasing trend, P = 0.552 for any trend), and for balanced data with additional adjustment for age, history of cancer, gastrointestinal diseases and use of CYP2J2 inhibitors (adjusted estimated probabilities from a logistic model) (t = -0.63, df = 273, P = 0.529 for a linear trend, t = -1.10, df = 273, P = 0.272 for a quadratic trend). Symbols are point estimates (inserted are numerical values), bars are 95% confidence intervals. Denoted is also the number (N) of subjects *per* subset: absolute number for the raw data, weighed number for covariate balanced data. The latter is lower than the former, since weighting always results in a loss of precision (effective sample size < original sample size).

In this balanced (pseudo)population, prevalence of patients with 0-2, 2-5 or 6-8 variant alleles across the *ABCB1* polymorphisms was similar in cases and controls: 30.5% *vs*. 30.8% (d = -0.007) for 0-2 variant alleles (no variant homozygous loci), 54.2% *vs*. 47.3% (d = 0.140) for 2-5 variant alleles, and 15.3% *vs*. 21.9% (d = -0.172) for 6-8 variant alleles (Table 4), just as was the case considering raw (unbalanced) data (Table 2). In reverse, Figure 1 shows proportions of cases across the patient subsets based on the increasing load of variant alleles across the four *ABCB1* polymorphisms: raw proportions, weighted proportions based on balanced data and estimated probabilities from a multivariable logistic model for balanced data with additional adjustment for age, history of cancer, gastrointestinal diseases and use of CYP2J2 inhibitors – there is no obvious trend in the proportion of cases (bleeding) with increasing number of variant alleles.

We identified 15 out of 16 possible haplotypes. The completely variant (c.1236T_c.2677T(A)_ c.3435T_c.2482-2236A) and completely wild-type (C_G_C_G) haplotypes by far prevailed (40.7% and 39.5%, respectively), with closely similar prevalence of cases (patients who experienced bleeding) (21.1% *vs.* 23.1%, respectively). All other haplotypes were observed with frequencies from 0.2% to 6.4% (cumulatively 19.8%), with 18% prevalence of cases (not shown). We observed 34 genotype combinations across the four polymorphisms (c.1236C>T, c.2677G>T(A), c.3435C>T, c.2482-2236G>A), most common of which was one with 4 variant alleles (*CT/GT(A)/CT/GA*, N = 82, 29.3%), followed by the completely wild-type combination (CC/GG/CC/GG, N = 53 (18.9%) and the completely variant combination (TT/TT(A) /TT/AA, N = 49, 17.5%). Prevalence of cases was closely similar - 20.7% *vs.* 26.4% *vs.* 22.4%, respectively. All other genotype combinations were observed with frequencies between 0.3% to 6.8% (cumulative N = 96, 44.3%), with cumulative prevalence of cases of 18% (not shown).

## Discussion

The purpose of pharmacogenomics research is to identify genetic traits that could be used as *a prirori* indicators of clinically relevant risks of failures or adverse effects of particular treatments in individual patients. With respect to this ultimate goal, the present analysis suggests that the four common *ABCB1* polymorphisms (rs1128503, rs2032582, rs1045642, rs4148738) – by far the most prevalent of all *ABCB1* polymorphisms in White Europeans – do not seem to have a clinically relevant effect on the risk of bleeding over the initial 6-12 months in the *de novo* rivaroxaban-treated patients. The study was conceived on the following grounds: (i) a reasonably expected incidence of bleeding of 20% over the first 6 months (*33*); (ii) the theoretical background suggesting that an increasing “load” of variant alleles across these polymorphisms could be reasonably expected to result in reduced ABCB1 function, *ie.*, increased rivaroxaban bioavailability and anticoagulant effect; (iii) we considered that “clinical relevance” might be indicated by a robust trend of increased risk of bleeding, such that the rate of 20% is resultant to a low rate (*e.g.,* 10%) with no or only a few variant alleles across the 4 polymorphisms, and then higher (*e.g.,* 20%, 30%) with higher number of variant alleles (*18*). Under such circumstances, the fact that we observed consistently similar risks of bleeding and no indication of any trend in patients with different “variant loads” (raw data, fully adjusted analysis), similar risks with “fully wild-type” and “fully variant” haplotypes, and with “fully variant” and “fully wild-type” genotype combinations across the four polymorphisms, justifies a conclusion about the lack of signal of any “practically relevant” effect.

The reports on the *ABCB1* polymorphisms and the risk of bleeding (major or clinically relevant non-major bleeding (CRNMB)) have thus far yielded controversial results. Comparison of outcomes in observational studies, particularly those involving genetic traits, is considerably less straightforward than in the case of randomized experiments because apparent differences or similarities could be due to many reasons other than the true underlying biological phenomenon that is actually evaluated, *e.g.,* chance and sample size (small samples and/or low number of events are highly susceptible to chance findings), target population and the sample particulars, study design, ethnic and geographical determinants, control of confounding and susceptibility to other biases imminent in observational data. In a study based on administrative data, 999 Finnish patients newly started on rivaroxaban due to non-valvular atrial fibrillation (NVAF), vascular disease, pulmonary embolism (PE), deep venous thrombosis (DVT) or cerebrovascular incidents (CVI), were followed-up over a median period of 4 months (*40*). With adjustment for concomitant antiplatelet use, wild-type patients and variant carriers at each of the four *ABCB1* polymorphisms (rs1128503, rs2032582, rs1045642, rs4148738) had a virtually identical risk of major or non-major clinically relevant bleeding, and the same was observed in the haplotype analysis (*40*). However, there were only 26 events – a few events more or less among either patient stratum (which could have occurred with essentially identical probability as the observed numbers), could have substantially changed the results (*40*). In a sample of 95 rivaroxaban-treated Chinese NVAF patients followed-up over one year, raw prevalence of genotypes at *ABCB1* polymorphisms rs1128503, rs1045642 and rs4148738 was closely similar between those who experienced bleedings and those who did not – but there were only 16/95 patients with bleeding events (*41*). Obviously, data is extremely fragile. Likewise, a report that included 155 NVAF patients of Mongolian descent started on rivaroxaban, reported similar raw prevalence of genotypes at *ABCB1* polymorphisms rs1128503, rs1045642 and rs4148738 in patients who experienced bleeding over the initial 7-10 days of treatment and in those who did not, but there were only 24 of the former, and the observational period was extremely short (*42*). In a cross-sectional study in 128 Russian NVAF patients older than 80 years of age with at least 7 days of treatment with rivaroxaban, 23 had a history of CRNMB (*43*). The authors report higher raw proportion of “bleeders” among rs1045642 variant homozygotes (12/41) *vs.* wild-type subjects (1/22), and among rs4148738 variant homozygotes (11/28) *vs*. wild-type subjects (3/37) – however, no control of confounding was undertaken, and the number of subjects and events (particularly among “wild-type” patients) was extremely low (*43*). Largely opposite results were reported in another study in Russian patients: 100 subjects with acute coronary syndrome and NVAF were treated with rivaroxaban and dual antiplatelet treatment for up to 12 months, and 38 developed major bleeding or CRNMB (*44*). The authors report an increased risk of bleeding as unadjusted (raw) odds ratio for rs1045642 wild-type subjects *vs.* variant carriers (exactly the opposite from the report in NVAF patients) of 3.13 (95%CI 1.03-9.52) (*43*). However, in addition to the fact of no confounding control, the authors erred in the calculation of the odds ratio – there were 13 “bleeders” among 29 wild-type subjects and 25 “bleeders” among 71 variant carriers, which gives OR = 1.50 (95%CI 0.62-3.58, P = 0.370). The authors also report unadjusted OR for rs4148738 variant carriers *vs.* wild-type subjects of 7.08 (95%CI 2.17 -23.1), but again err in the odds ratio calculation: there were 5 “bleeders” among 18 wild-type patients, and 33 “bleeders” among 82 variant carriers which gives OR = 1.75 (95%CI 0.57-5.38, P = 0.328) (we calculated Mantel-Haenszel ORs and CIs, and respective test statistics) (*44*).

Three further studies could be considered less informative on the topic, because they jointly considered patients treated with different NOACs. A single-centre registry analysis in the USA included 2364 White outpatients with NVAF followed-up over 1-3 years (*45*). With a comprehensive confounding control, the risk of major/CRNM bleeding was closely similar in wild-type patients and variant carriers at rs1128503, rs2032582, and rs1045642 considered individually and as haplotypes, but patients treated with rivaroxaban (N = 802) and apixaban (N = 1324) were considered jointly (*45*). Similarly, a cross-sectional case-control study in Korean patients (50 with bleeding and 418 controls) jointly considered patients on apixaban, edoxaban, dabigatran and rivaroxaban (N = 74), and suggested no univariate association between the case status and *ABCB1* polymorphisms rs1128503, rs2032582 or rs1045642 (*46*). In contrast, another cross-sectional study in Korean patients (64 cases and 229 controls) treated with either apixaban or rivaroxaban (numbers not reported) suggested higher risk in variant carriers at rs1045642 *vs.* wild-type subjects (*47*). However, the reported OR (3.2, 1.35-7.43) was derived from a model with 10 covariates and was likely severely biased away from the null, since there were only 64 cases, and no measures were undertaken to reduce the bias (*48*).

The obvious limitation of the present study is a moderately-sized single-centre sample. However, from the purely “technical” standpoint, by using energy balancing to control for 18 of the 22 plausibly relevant confounders, we achieved a situation in which the final logistic model with 5 independents and effective sample of 58 cases and 205 controls enabled us to generate estimated probabilities of bleeding across the levels of *ABCB1* variant allele “loads” reasonably protected from bias inherent to logistic models with a limited number of events. The confounders that we accounted for comprehensively addressed demographic, comorbidity, co-medication and genetic factors, and were observed and captured in real-time. As supported by the HWE tests, classification of patients regarding the *ABCB1* and other polymorphisms was most likely correct, and the prospective study design ascertained adequate classification of “cases” and “controls”, particularly since all controls had at least 7 and a maximum of 10 months of follow-up without bleeding. We limited the “case” designation to patients who experienced bleeding within the initial 6 months, thus minimizing the interference of post-baseline (intercurrent) events that might have been difficult to control. The reasoning that if some genetic trait indeed has an important impact on the bleeding risk, this should be obvious already over the first 6 months of treatment is also medically justified: in the cited Finnish study, practically all bleeding events occurred within the initial 6 months (*40*).

Finally, our approach of categorization of patients with respect to their genotypes across the four strongly linked polymorphisms based on the “load” (number) of variant alleles might be objected. However, we find it to be biologically plausible – it reflects the underlying rationale of (presumably) altered ABCB1 transporter function consequent to the presence of variant alleles: subjects classified as those with 0 (wild type) to 2 variant alleles (out of 8 possible) but with no variant homozygosity could be reasonably viewed as those with no or minimal (hypothetical) alterations of the transporter functions, whereas those with 6 to 8 such alleles (all four loci variant homozygous) could be viewed as those with maximal (hypothetical) alterations. Even if variant alleles on different loci “favored” different (hypothetical) effects, it is the “net” effect of the entire set-up across the four polymorphisms that would be informative: an increasing trend in bleeding incidence across such a “variant gradient” would indicate their practical relevance. The fact of similar raw and weighted (adjusted) prevalence of genotypes at each of the four polymorphisms between cases and controls, and comparable probabilities of bleeding in the haplotype analysis and analysis of genotype combinations support the main observations.

In conclusion, the present nested case-control study strongly suggests that in White Europeans the common coding *ABCB1* polymorphisms rs1128503 (c.1236C>T), rs2032582 (c.2677G>T/A), rs1045642 (c.3435C>T) and rs4148738 (2482-2236G>A) do not have any clinically relevant effect on the risk of bleeding over the initial 6 months in rivaroxaban-treated patients.

## Data Availability

The data presented in this study are available from the corresponding author on reasonable request.

## References

[1] Yan VKC Li HL Wei L Knapp MRJ Wong ICK Chan EW. Evolving Trends in Consumption of Direct Oral Anticoagulants in 65 Countries/Regions from 2008 to 2019. Drugs. 2023;83:315–40. 10.1007/s40265-023-01837-0 36840892

[2] Draganić P, Oštarčević S, Škribulja M. The Report on Drug Utilisation in the Republic of Croatia 2017 - 2021. Agency for Medicines and Medical Products – HALMED. 2022. (in Croatian)

[3] Chen A Stecker E Warden BA. Direct Oral Anticoagulant Use: A Practical Guide to Common Clinical Challenges. J Am Heart Assoc. 2020;9:e017559. 10.1161/JAHA.120.017559 32538234 PMC7670541

[4] Verdecchia P Angeli F Aita A Bartolini C Reboldi G. Why switch from warfarin to NOACs? Intern Emerg Med. 2016;11:289–93. 10.1007/s11739-016-1411-0 26972708

[5] Diep R Garcia D. Should we monitor the direct oral anticoagulants? J Thromb Thrombolysis. 2020;50:30–2. 10.1007/s11239-020-02119-2 32323189 PMC7433889

[6] Chen A Stecker E. A Warden B. Direct Oral Anticoagulant Use: A Practical Guide to Common Clinical Challenges. J Am Heart Assoc. 2020;9:e017559. 10.1161/JAHA.120.017559 32538234 PMC7670541

[7] Mueck W Stampfuss J. Kubitza, Becka M. Clinical Pharmacokinetic and Pharmacodynamic Profile of Rivaroxaban. Clin. Pharmacokinet. 2014;53:1–16. 10.1007/s40262-013-0100-7 23999929 PMC3889701

[8] Ferri N Colombo E Tenconi M Baldessin L Corsini A. Drug-Drug Interactions of Direct Oral Anticoagulants (DOACs): From Pharmacological to Clinical Practice. Pharmaceutics. 2022;14:1120. 10.3390/pharmaceutics14061120 35745692 PMC9229376

[9] Shnayder NA Petrova MM Shesternya PA Savinova AV Bochanova EN Zimnitskaya OV Using Pharmacogenetics of Direct Oral Anticoagulants to Predict Changes in Their Pharmacokinetics and the Risk of Adverse Drug Reactions. Biomedicines. 2021;9:451. 10.3390/biomedicines9050451 33922084 PMC8143539

[10] Kanuri SH Kreutz RP. Pharmacogenomics of Novel Direct Oral Anticoagulants: Newly Identified Genes and Genetic Variants. J Pers Med. 2019;9:7. 10.3390/jpm9010007 30658513 PMC6463033

[11] Raymond J Imbert L Cousin T Duflot T Varin R Wils J Pharmacogenetics of Direct Oral Anticoagulants: A Systematic Review. J Pers Med. 2021;11:37. 10.3390/jpm11010037 33440670 PMC7826504

[12] Lin JH Yamazaki M. Role of P-glycoprotein in pharmacokinetics: clinical implications. Clin Pharmacokinet. 2003;42:59–98. 10.2165/00003088-200342010-00003 12489979

[13] Fromm MF. Importance of P-glycoprotein at blood-tissue barriers. Trends Pharmacol Sci. 2004;25:423–9. 10.1016/j.tips.2004.06.002 15276711

[14] Brinkmann U Eichelbaum M. Polymorphisms in the ABC drug transporter gene MDR1. Pharmacogenomics J. 2001;1:59–64. 10.1038/sj.tpj.6500001 11913728

[15] Woodahl EL Ho RJ. The role of MDR1 genetic polymorphisms in interindividual variability in P-glycoprotein expression and function. Curr Drug Metab. 2004;5:11–9. 10.2174/1389200043489108 14965248

[16] Leschziner GD Andrew T Pirmohamed M Johnson MR. ABCB1 Genotype and PGP Expression, Function and Therapeutic Drug Response: A Critical Review and Recommendations for Future Research. Pharmacogenomics J. 2007;7:154–79. 10.1038/sj.tpj.6500413 16969364

[17] Tulsyan S Mittal RD Mittal B. The effect of ABCB1 polymorphisms on the outcome of breast cancer treatment. Pharmgenomics Pers Med. 2016;9:47–58. 10.2147/PGPM.S86672 27175090 PMC4854269

[18] Gschwind L Rollason V Daali Y Bonnabry P Dayer P Desmeules JA. Role of P-glycoprotein in the uptake/efflux transport of oral vitamin K antagonists and rivaroxaban through the Caco-2 cell model. Basic Clin Pharmacol Toxicol. 2013;113:259–65. 10.1111/bcpt.12084 23663291

[19] Gong IY Mansell SE Kim RB. Absence of both MDR1 (ABCB1) and breast cancer resistance protein (ABCG2) transporters significantly alters rivaroxaban disposition and central nervous system entry. Basic Clin Pharmacol Toxicol. 2013;112:164–70. 10.1111/bcpt.12005 22958812

[20] Sennesael AL Panin N Vancraeynest C Pochet L Spinewine A Haufroid V Effect of ABCB1 genetic polymorphisms on the transport of rivaroxaban in HEK293 recombinant cell lines. Sci Rep. 2018;8:10514. 10.1038/s41598-018-28622-4 30002384 PMC6043481

[21] Martin FJ Amode MR Aneja A Austine-Orimoloye O Azov AG Barnes I Ensembl 2023, release 110. Nucleic Acids Res. 2023;51:D933–41. 10.1093/nar/gkac958 36318249 PMC9825606

[22] Fung KL Gottesman MM. A synonymous polymorphism in a common MDR1 (ABCB1) haplotype shapes protein function. Biochim Biophys Acta. 2009;1794:860–71. 10.1016/j.bbapap.2009.02.014 19285158 PMC2810319

[23] Leschziner GD Andrew T Pirmohamed M Johnson MR. ABCB1 genotype and PGP expression, function and therapeutic drug response: a critical review and recommendations for future research. Pharmacogenomics J. 2007;7:154–79. 10.1038/sj.tpj.6500413 16969364

[24] Ing Lorenzini K Daali Y Fontana P Desmeules J Samer C. Rivaroxaban-Induced Hemorrhage Associated with ABCB1 Genetic Defect. Front Pharmacol. 2016;7:494. 10.3389/fphar.2016.00494 28066243 PMC5165251

[25] Lexicomp Drug Interactions. UpToDate, Inc. Available from: https://www.uptodate.com/drug-interactions/#didruglist. Accessed September 1, 2023.

[26] Schulman S Kearon C Subcommittee on Control of Anticoagulation of the Scientific and Standardization Committee of the International Society on Thrombosis and Haemostasis. Definition of major bleeding in clinical investigations of antihemostatic medicinal products in non-surgical patients. J Thromb Haemost. 2005;3:692–4. 10.1111/j.1538-7836.2005.01204.x 15842354

[27] Kaatz S Ahmad D Spyropoulos AC Schulman S Subcommittee on Control of Anticoagulation. Definition of clinically relevant non-major bleeding in studies of anticoagulants in atrial fibrillation and venous thromboembolic disease in non-surgical patients: communication from the SSC of the ISTH. J Thromb Haemost. 2015;13:2119–26. 10.1111/jth.13140 26764429

[28] Šimičević L Slišković AM Kirhmajer MV Ganoci L Holik H Palić J Risk Factors for Rivaroxaban-Related Bleeding Events-Possible Role of Pharmacogenetics: Case Series. Pharmacy (Basel). 2023;11:29. 10.3390/pharmacy11010029 36827667 PMC9966833

[29] Arjomand-Nahad F Diefenbach K Landt O Gaikovitch E Roots I. Genotyping of the triallelic variant G2677T/A in MDR1 using LightCycler with locked-nucleic-acid-modified hybridization probes. Anal Biochem. 2004;334:201–3. 10.1016/j.ab.2004.07.030 15464971

[30] Elens L van Gelder T Hesselink DA Haufroid V van Schaik RH. CYP3A4*22: promising newly identified CYP3A4 variant allele for personalizing pharmacotherapy. Pharmacogenomics. 2013;14:47–62. 10.2217/pgs.12.187 23252948

[31] Polonikov AV Ponomarenko IV Bykanova MA Sirotina SS Bocharova AV Vagaytseva KV A comprehensive study revealed SNP-SNP interactions and a sex-dependent relationship between polymorphisms of the CYP2J2 gene and hypertension risk. Hypertens Res. 2019;42:257–72. 10.1038/s41440-018-0142-1 30518987

[32] Spiecker M Darius H Hankeln T Soufi M Sattler AM Schaefer JR Risk of coronary artery disease associated with polymorphism of the cytochrome P450 epoxygenase CYP2J2. Circulation. 2004;110:2132–6. 10.1161/01.CIR.0000143832.91812.60 15466638 PMC2633457

[33] Schaefer JK Errickson J Kong Y Ali MA Chiplakatti N Hamart B A comparison of bleeding events among patients on apixaban, rivaroxaban, and warfarin for atrial fibrillation and/or venous thromboembolism. Blood. 2023;142 suppl 1:135. 10.1182/blood-2023-184457

[34] Nam JM. Simple approximation for calculating sample sizes for detecting linear trend in proportions. Biometrics. 1987;43:701–5. 10.2307/2532006 3663825

[35] Greifer N. WeightIt: Weighting for Covariate Balance in Observational Studies. Available from: https://CRAN.R-project.org/package=WeightIt. Accessed May 22, 2023.

[36] R Core Team. R: A language and environment for statistical computing. R Foundation for Statistical Computing, 2020. Vienna, Austria. Available from: https://www.R-project.org. Accessed May 22, 2023.

[37] Huling JD Greifer N Chen G. Independence weights for causal inference with continuous treatments. J Am Stat Assoc. 2023. 10.1080/01621459.2023.2213485

[38] Huling JD, Mak S. Energy balancing of covariate distributions. arXiv:2004.13962v5 (stat.ME) [Preprint]. 2022 [cited 2023 May 22]. Available from: https://doi.org/10.48550/arXiv.2004.13962

[39] Gaunt TR Rodriquez S Day INM. Cubic exact solution for the estimation of pairwise haplotype frequencies: implications for linkage disequlibrium analysesand a web tool ‘CubeX’. BMC Bioinformatics. 2007;8:428. 10.1186/1471-2105-8-428 17980034 PMC2180187

[40] Lähteenmäki J Vuorinen AL Pajula J Harno K Lehto M Niemi M Pharmacogenetics of Bleeding and Thromboembolic Events in Direct Oral Anticoagulant Users. Clin Pharmacol Ther. 2021;110:768–76. 10.1002/cpt.2316 34043814

[41] Wu T Wu S Li L Xiang J Wang N Chen W The impact of ABCB1, CYP3A4/5 and ABCG2 gene polymorphisms on rivaroxaban trough concentrations and bleeding events in patients with non-valvular atrial fibrillation. Hum Genomics. 2023;17:59. 10.1186/s40246-023-00506-3 37420302 PMC10327396

[42] Wang Y Chen M Chen H Wang F. Incluence of ABCB1 gene polymorphisms on rivaroxaban blood concentration and hemorrhagic events in patients with atrial fibrillation. Front Pharmacol. 2021;12:639854. 10.3389/fphar.2021.639854 33935730 PMC8079976

[43] Sychev D Ostroumova O Cherniaeva M Shakhgildian N Mirzaev K Abdullaev S The influence of ABCB1 (rs1045642 and rs4148738) gene polymorphisms on rivaroxaban pharmacokinetics in patients aged 80 years and older with nonvalvular atrial fibrillation. High Blood Press Cardiovasc Prev. 2022;29:469–80. 10.1007/s40292-022-00536-3 35960493

[44] Baturina O Chashkina M Andreev D Mirzaev K Bykova A Suvorov A Pharmacokinetic and pharmacogenetic predictors of major bleeding events in patients with an acute coronary syndrome and atrial fibrillation receiving combined antithrombotic therapy. J Pers Med. 2023;13:1371. 10.3390/jpm13091371 37763139 PMC10532904

[45] Campos-Staffico AM Dorsch MP Barnes GD Zhu HJ Limdi NA Luzum JA. Eight pharmacokinetic genetic variants are not associated with the risk of bleeding from direct oral anticoagulatns in non-valvular atrial fibrillation patients. Front Pharmacol. 2022;13:1007113. 10.3389/fphar.2022.1007113 36506510 PMC9730333

[46] Yoon HY Song TJ Yee J Park J Gwak HS. Association between Genetic Polymorphisms and Bleeding in Patients on Direct Oral Anticoagulants. Pharmaceutics. 2022;14:1889. 10.3390/pharmaceutics14091889 36145636 PMC9501033

[47] Kim H Song TJ Yee J Kim DH Park J Gwak HS. ABCG2 Gene Polymorphisms May Affect the Bleeding Risk in Patients on Apixaban and Rivaroxaban. Drug Des Devel Ther. 2023;17:2513–22. 10.2147/DDDT.S417096 37641689 PMC10460569

[48] Rainey C McCaskey K. Estimating logit models with small samples. Political Sci Res Methods. 2021;9:549–64. 10.1017/psrm.2021.9

